# Abundant Neural circRNA Cdr1as Is Not Indispensable for Retina Maintenance

**DOI:** 10.3389/fcell.2020.565543

**Published:** 2020-11-06

**Authors:** Xue-Jiao Chen, Meng-Lan Li, Ya-Han Wang, Hao Mou, Zhen Wu, Siqi Bao, Ze-Hua Xu, Hang Zhang, Xiao-Yun Wang, Chang-Jun Zhang, Xiangyang Xue, Zi-Bing Jin

**Affiliations:** ^1^School of Ophthalmology and Optometry and Eye Hospital, School of Biomedical Engineering, Wenzhou Medical University, Wenzhou, China; ^2^School of Basic Medical Science, Wenzhou Medical University, Wenzhou, China; ^3^Beijing Ophthalmology and Visual Sciences Key Laboratory, Beijing Institute of Ophthalmology, Beijing Tongren Eye Center, Beijing Tongren Hospital, Capital Medical University, Beijing, China

**Keywords:** retina, circular RNA, CDR1as, retinal function, knockout

## Abstract

Cdr1as is the abundant circular RNA (circRNA) in human and vertebrate retinas. However, the role of Cdr1as in the retina remains unknown. In this study, we aimed to generate a Cdr1as knockout (KO) mouse model and investigate the retinal consequences of Cdr1as loss of function. Through *in situ* hybridization (ISH), we demonstrated that Cdr1as is mainly expressed in the inner retina. Using CRISPR/Cas9 targeting Cdr1as, we successfully generated KO mice. We carried out ocular examinations in the KO mice until postnatal day 500. Compared with the age-matched wild-type (WT) siblings, the KO mice displayed increased b-wave amplitude of photopic electrophysiological response and reduced vision contrast sensitivity. Through small RNA profiling of the retinas, we determined that miR-7 was downregulated, while its target genes were upregulated. Taken together, our results demonstrated for the first time that Cdr1as ablation led to a mild retinal consequence in mice, indicating that Cdr1as abundance is not indispensable for retinal development and maintenance.

## Introduction

Circular RNAs (circRNAs) are a class of stable, covalently closed RNA molecules produced from precursor mRNAs (pre-mRNAs) through back-splicing reactions (Jeck et al., 2013; Lasda and Parker, 2014; Ebbesen et al., 2017). Recently, a large number of circRNAs with complex tissue- and stage-specific expression patterns have been identified in eukaryotes (Salzman et al., 2013; Guo et al., 2014; Veno et al., 2015). Recent studies have demonstrated the biological functions of circRNAs at the molecular level (Chen, 2016; Li et al., 2018), including sequestration of microRNAs or associated proteins (Hansen et al., 2013; Li et al., 2017), splicing interference of their linear cognates (Ashwal-Fluss et al., 2014), modulation of transcription of parent genes (Li et al., 2015), and translation to produce peptides (Wang and Wang, 2015). However, considering the low efficiency of back-splicing and special structure of circRNAs, the function of most individual circRNAs remains elusive.

Circular RNAs are reported to be highly enriched in the central nervous system (CNS) and regulate synaptic function (Rybak-Wolf et al., 2015; You et al., 2015). As the most extensively characterized circRNA, Cdr1as is highly abundant in neurons and acts as a post-transcriptional regulator with many conserved binding sites for miR-7 and miR-671 (Hansen et al., 2013; Memczak et al., 2013). Expression of human Cdr1as in zebrafish caused midbrain defects, similar to miR-7 knockdown, and Cdr1as was once considered to possess important regulatory function (Memczak et al., 2013). However, Cdr1as knockout (KO) mouse, the first and sole circRNA KO mouse model, displayed a mild neuropsychiatric phenotype reflected in sensorimotor gating deficit and dysfunctional synaptic transmission (Piwecka et al., 2017).

The retina is a key part of the CNS and is responsible for vision production. As the abundant circRNA in the retina, Cdr1as is upregulated during retinal development (Chen et al., 2020), indicating the regulatory potential of Cdr1as in the retina. Whether and how Cdr1as affects retinal function remains unknown. To address this question, we generated a Cdr1as KO model using the CRISPR/Cas9 strategy. We found that Cdr1as deletion in mice caused mild alterations both in retinal phenotypic and vision functions as late as P300. In addition, miR-7, its target genes, and immediate early genes (IEGs) were deregulated in the Cdr1as KO retina. Our results demonstrated for the first time that Cdr1as ablation led to a mild retinal consequence in mice, indicating that Cdr1as abundance is not indispensable for retinal development and maintenance.

## Results

### Spatial Expression Pattern of Cdr1as in Retina and Other Tissues

It has been reported that Cdr1as is highly abundant in neurons, but is scarce in other tissues (Piwecka et al., 2017). To validate its spatial expression pattern, we investigated the expression of Cdr1as in different tissues by quantitative RT-PCR (qRT-PCR). As expected, Cdr1as was highly expressed in the brain and retina, and was expressed at low levels in other tissues, such as the lung, heart, liver, spleen, kidney, and muscle (Supplementary Figure 1A). To evaluate the localization of Cdr1as, we performed *in situ* hybridization (ISH) in the mouse retina at P120 using the BaseScope assay (Supplementary Figure 1B). We found Cdr1as located in the inner retina, predominantly in the inner nuclear layer (INL) near the inner plexiform layer (IPL). ISH and immunofluorescence (IF) staining demonstrated a portion of Cdr1as expressed in TH+ amacrine cells, which could release dopamine neurotransmitter (Supplementary Figure 1C). Together with our previous findings that Cdr1as increased sharply from P1 to P14 (Supplementary Figure 1D), these results indicated the potential role of Cdr1as during retinal development. However, we could not find any obvious abnormal phenotype during the retinal development, these may due to the compensation mechanisms in organisms.

### Successful Knockout of Cdr1as in Mice

**FIGURE 1:**
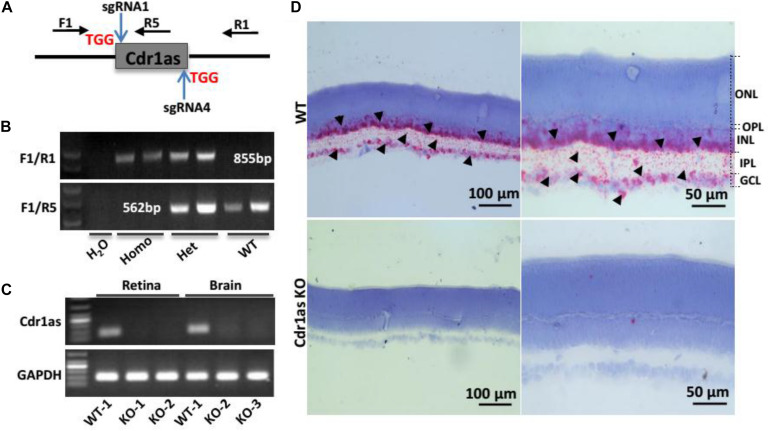
Generation and identification of Cdr1as KO mice. **(A)** Cdr1as was removing using CRISPR/Cas9 strategy, and two sgRNAs targeting different regions of Cdr1as were designed. PAM sequences were labeled in red. **(B)** Genotyping strategy for the WT, Hete, and Homo strains using F1/R1 and F1/R5, which are shown in **(A)**. **(C)** QPCR analysis of Cdr1as expression in the retinas and brains of WT and Cdr1as KO mice at P30. **(D)** BaseScope assay showing the expression of Cdr1as in WT and Cdr1as KO retinas at P30; red dots show the sense of ISH image, which has been labeled by black arrowhead. ONL, outer nuclear layer; OPL, outer plexiform layer, INL, inner nuclear layer; IPL, inner plexiform layer; GCL, ganglion cell layer.

To investigate the role of Cdr1as in the retina, we attempted to generate KO mice by using CRISPR/Cas9. Because the linear transcript of Cdr1as cannot be detected, it is feasible to explore the function of Cdr1as using a KO mouse model without concerning the phenotype caused by linear transcript interruption. In this study, we used the CRISPR/Cas9 strategy to generate the Cdr1as KO mouse model. Two sgRNAs were designed to bind upstream and downstream of Cdr1as splice sites (Figure 1A and Supplementary Table 1). The F1/R1 and F1/R5 primer pairs capable of distinguishing homozygotes from the heterozygotes and wild-type (WT) were designed for genotyping (Figure 1B and Supplementary Table 2). The head-to-tail junction sequence was confirmed by Sanger sequencing (Supplementary Figure 2). qRT-PCR experiments showed that Cdr1as disappeared in retinal and brain tissues of KO mice (Figure 1C). Additionally, ISH further validated the successful deletion of Cdr1as in the retina of KO mice (Figure 1D). The overall survival and life span of mice revealed no difference between Cdr1as KO and WT mice.

### Cdr1as Ablation Does Not Alter Retinal Structure

**FIGURE 2:**
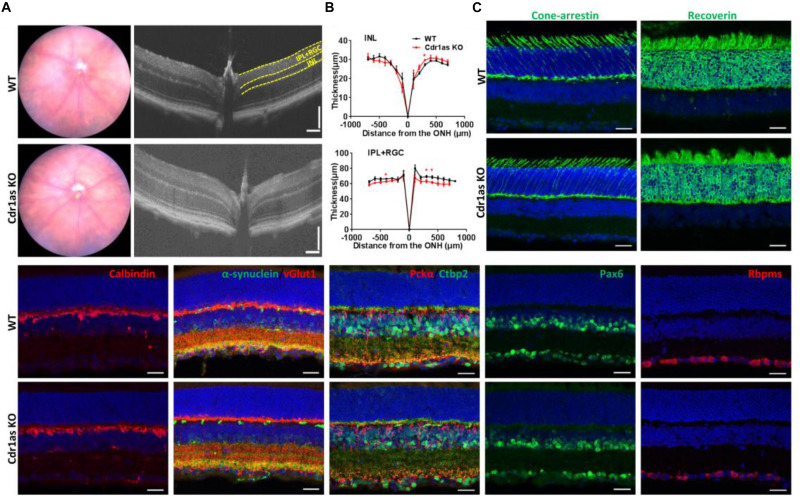
Structural phenotypes of retina in the adult Cdr1as KO mice. **(A)** Fundus photographs of WT and Cdr1as KO retinas in adult (*n* = 4) (left), OCT examination of WT and Cdr1as KO retinas at P70 (right). **(B)** Quantification of the thickness of the INL and IPL+RGC from the SD-OCT image in **(A)** (*n* = 5 for WT, *n* = 4 for KO). **(C)** Immunostaining of retinal neurons and synapses in WT and Cdr1as retinas (*n* = 3, scale bar: 25 μm).

To assess the retinal structure in Cdr1as KO mice, fundus photography and high-resolution spectral-domain optical coherence tomography (SD-OCT) were performed to detect the retinal morphologies and organization in adult and aged mice. Fundus photographs showed normal fundus appearances in both WT and KO mice at P70, P360, and P500 (Figure 2A and Supplementary Figure 3A). *In vivo* OCT imaging displayed that the inner retinal layer was unaltered obviously, even though a few changes happened at certain locations in Cdr1as KO mice (Figure 2B and Supplementary Figures 3B–D). We further examined the photoreceptor, retinal neurons, and synaptic structure by IF staining in adult mice. Photoreceptor cells, immunolabeled for recoverin and cone-arrestin, showed a preserved pattern in Cdr1as KO as in WT retinas. Consistent with this, immunostaining for calbindin, pkcα, pax6, and rbpms to label horizontal cells, bipolar cells, amacrine cells, and ganglion cells were also unchanged with the Cdr1as deletion. Additionally, immunostaining patterns for synaptic vGlut1, α-synuclein, and ctbp2 demonstrated similar patterns in the plexiform layer (Figure 2C and Supplementary Figures 4A–C).

### Cdr1as Knockout Resulted in Slight Changes of Retinal Function

**FIGURE 3:**
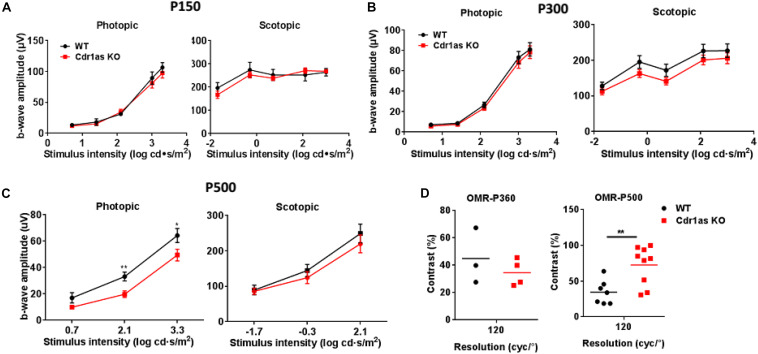
Assessment of retinal function in the adult and aged Cdr1as KO mice. **(A)** b-Wave amplitudes of scotopic and photopic for the Cdr1as KO and WT retinas at P150 (*n* = 5 for WT, *n* = 6 for KO). **(B)** b-Wave amplitudes of scotopic and photopic for the Cdr1as KO and WT retinas at P300 (*n* = 13 for WT, *n* = 13 for KO). **(C)** b-Wave amplitudes of scotopic and photopic for the Cdr1as KO and WT retinas at P500 (*n* = 10 for WT, *n* = 10 for KO). **(D)** OMR examination of Cdr1as KO and WT mice at P360 (*n* = 3 for WT, *n* = 4 for KO) and P500 (*n* = 7 for WT, *n* = 9 for KO). The normalized values represent means ± SEM. *P < 0.05, **P < 0.01, Student’s *t*-test.

To investigate the impact of Cdr1as deletion on visional function, we first monitored electroretinography (ERG) responses in adult and aged mice. The b wave amplitudes of both scotopic and photopic ERG response were unchanged at P150 and P300 (Figures 3A,B). However, ERG results of elder mice (P500) showed that the photopic b wave amplitude increased significantly (Figure 3C). This phenotype is similar to the effects mediated by dopamine D2 receptor (D2R) KO in mice (Lavoie et al., 2014; Tian et al., 2015) and D2R antagonist in goldfish (Kim and Jung, 2012) and cats (Schneider and Zrenner, 1991). Indicating the function of dopamine impaired in Cdr1as KO mice. Due to partial Cdr1as expressed in certain amacrine cells, which could release dopamine. Cdr1as deletion results in abnormal function of amacrine and then further affects dopamine function. The major ERG wave components, oscillatory potentials (OPS), which are displayed by certain amacrine cells in the retina were also analyzed at P240. As a result, there was no significantly difference between WT and KO mice, even though the slight lower amplitude of OPS was observed in Cdr1as KO mice (Supplementary Figure 3E). Considering that Cdr1as is expressed in the inner retina, the visual evoked potential (VEP) was applied to assess the electric conduction from the retina to visual cortices at P240 and P500. The amplitudes from the N1 to P1 peak in Cdr1as KO mice were slightly decreased, but no difference was observed in two groups (Supplementary Figure 3F).

Additionally, an optomotor response (OMR) assay was performed to assess the animal photopic response to a visual stimulus at P360 and P500. We found that both Cdr1as KO and WT mice responded to the rotating grating through head tracking, the vision contrasts sensitivity showed no significant alteration at P360 but decreased significantly at P500 (Figure 3D). The reduced vision acuity may be due to the reduction of dopamine in the retina, which has been reported previously (Witkovsky, 2004). Taken together, these results indicated that the deletion of Cdr1as has a very mild effect on visual function.

### Retinal miRNAs and Their Target Genes Are Deregulated in Cdr1as KO Retinas

**FIGURE 4:**
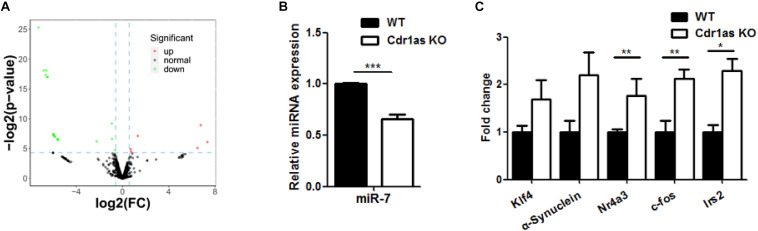
miRNAs and genes expression changes in Cdr1as KO retina. **(A)** Differentially expressed genes between Cdr1as and WT retinas in adult mouse (*n* = 3). The red dots represent the upregulated genes, and green dots represent the downregulated genes. **(B)** miR-7 expression changes in Cdr1as and WT retinas (*n* = 3). **(C)** Changes in the expression of target genes of miR-7 in Cdr1as and WT retinas (*n* = 3). The normalized values represent means ± SEM. *P < 0.05, **P < 0.01, ***P < 0.001, Student’s *t*-test.

Following the assessment of the retinal structure and function in Cdr1as KO mice, molecular phenotypes were further investigated. Considering the strong sponge efficiency of Cdr1as for miRNAs, we evaluated the expression pattern of retinal miRNAs in Cdr1as KO mice. Whole-retinal small RNA-seq from adult mice showed that the expression of 25 miRNAs, such as miR-7, miR-344c, miR-322, miR-326, miR-122, and miR-7048 was significantly changed (fold change > 1.5, *p* < 0.05) between the Cdr1as KO and WT groups (Figure 4A), miR-7, the highly expressed miRNA, was confirmed by qRT-PCR and was downregulated in Cdr1as KO retinas (Figure 4B). The miR-7 target genes we tested, including Nr4a3, Klf4, Irs2, α-synuclein, and c-fos, displayed an increasing expression tendency (Figure 4C). Among the target genes, Nr4a3 and c-fos are IEGs, which have been linked to neural activity and could respond to different stimuli (Caputto and Guido, 2000; Piwecka et al., 2017). Other IEGs, such as Egr1, Egr4, and Arc, were also upregulated in KO retinas (Supplementary Figure 5). These results suggested that degeneration in Cdr1as KO retinas was more likely to occur under certain stimuli.

**FIGURE 5:**
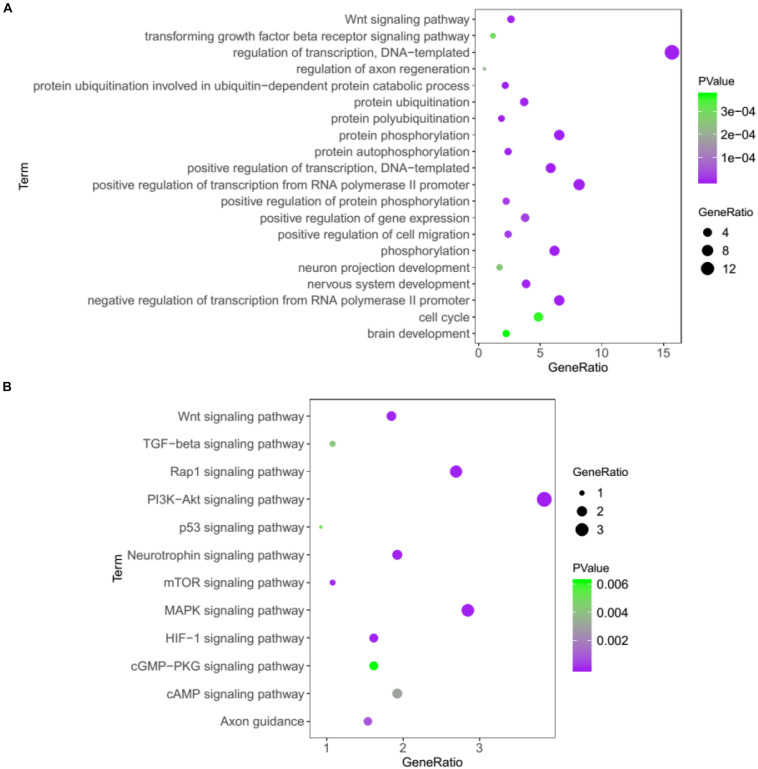
Bioinformatics analysis of the target genes of 25 miRNAs that changed significantly in Cdr1as KO retina using GO **(A)** and KEGG pathway analysis **(B)**.

In addition, we performed the GO and KEGG to analyze the 1686 target genes of 25 miRNAs that changed significantly. As was shown in Figure 5A, several GO terms were found to be significantly enriched, including nervous system development, regulation of axon regeneration, and neuron projection development, suggesting that some biological processes of the neuron system may be affected in the KO mice. The subsequent KEGG pathway analysis showed that PI3K-Akt, MAPK, Hif-1, Wnt, mTOR, and other important signaling pathways were significantly enriched (Figure 5B).

## Discussion

In this study, we successfully generated a Cdr1as KO mouse model and first explored the function of Cdr1as in the retina. Despite of the seemingly normal appearance, Cdr1as KO mice had mild retinal phenotypes. We found that miR-7, which is highly sponged by Cdr1as, was downregulated and IEGs were upregulated (Supplementary Figure 5). These data suggested that Cdr1as KO retinas might be more vulnerable to degenerative alterations. The function of Cdr1as retina could be studied under different stimuli, such as light and circadian regulation.

The localization of Cdr1as was partially similar to that of α-synuclein (Surguchov et al., 2001; Chandra et al., 2005), which is aggregated in Parkinson’s disease retinas. miR-7 was reported to repress α-synuclein expression and protect cells against oxidative stress (Junn et al., 2009). We also found phospho-α-synuclein increased in Cdr1as KO retina (Supplementary Figure 6). Whether α-synuclein aggregation induced by miR-7 downregulation could cause the retina to be more vulnerable to stress needs further study.

Among the downregulated miRNAs in Cdr1as KO retinas, miR-326, miR-322, miR-344, miR-7048, and miR-122 should be mentioned here. CDC42 which is a member of the Rho GTPase family is a target of miR-326, and CDC42 dysregulation is linked to neuronal diseases such as Alzheimer’s and Parkinson’s disease (Zhu et al., 2000; Mitchell et al., 2007). CDC42 was upregulated in hippocampal neurons of Alzheimer’s patients (Zhu et al., 2000). Abnormal CDC42 may lead to altered retinal function. RNA helicase Ddx3x was found to be the direct target of miR-322 (Che et al., 2019). Ddx3x was reported to be essential for the even distribution of cells across layers, and Ddx3x was shown to be involved in the network that could control miR-183C accumulation in the photoreceptor layer architecture (Krol et al., 2015). Dysregulation of Ddx3x may contribute to the abnormalities in retinal function. miR-344 directly targets glycogen synthase kinase 3 beta (GSK3β) (Chen et al., 2014), which was reported to regulate Wnt signaling via phosphorylation of β-catenin (Cohen and Frame, 2001). Additionally, inactivation of GSK3β led to β-catenin stabilization and MG proliferation without retinal injury (Yao et al., 2016). miR-7048 and its target Ascl1 were involved in enhanced neuronal regeneration after injury (Lisi et al., 2017). miR-122 was downregulated in canine models of retinal degeneration (Kok et al., 2015). miR-122 was also reported to be downregulated in extracellular vesicles from ARPE19 cells under oxidative conditions (Genini et al., 2014). We speculate that dysregulated miRNA-induced target genes alteration could have some effects on the retina. We also predicted the targets of 25 miRNA that changed significantly using seven tools and further performed bioinformatics analysis based on these 1686 target genes. GO and KEGG pathway analysis showed that these genes were related to neuron system and several important signaling pathway. These results broadly reflected the biological effect of Cdr1as in the retina.

There are several unknowns in the present study. First, since the location of Cdr1as is mainly in the inner retina while the ERG response is derived from photoreceptors to bipolar and amacrine cells, it seems that ERG is not a reasonable method to assess the retinal function of Cdr1as KO retinas. In contrast, the VEP results in our study seemed to be more believable compared to the ERG data. Second, we do not know if any ultrastructural changes in the retina, which could be done by electron microscopy analysis in the future. Third, the abundant expression of Cdr1as in retina seems to be mismatch with the mild retinal phenotype in the Cdr1as KO mice.

As mentioned above, Cdr1as is the most abundant in the mammalian brain, followed by the retina and is expressed at least level or absent in other tissues. It has been reported that loss-of-function Cdr1as *in vivo* causes miRNA deregulation, mild physiological consequences, and impaired sensorimotor gating (Piwecka et al., 2017). In contrast, the investigation of Cdr1as in zebrafish experiments showed the potential regulatory role of Cdr1as (Memczak et al., 2013); these differences might be due to the different experimental approaches (Oltra et al., 2019). Additionally, the slight effect of Cdr1as on the brain and retina further confirmed that as epigenetic factors, the regulation of some circRNAs or miRNAs is typically relatively minor, even though their high expression in eukaryotes (Jin et al., 2009; Piwecka et al., 2017; Kleaveland et al., 2018).

Taken together, we demonstrated for the first time that Cdr1as is abundantly expressed in the inner retina and that its KO altered retinal miRNA expression patterns as well as the expression of their target genes but had a slight influence on retinal morphogenesis and function.

## Materials and Methods

### Animals

The Cdr1as KO mice and C57BL/6J mice were bred and maintained in the animal facility of Wenzhou Medical University with a 12-h light/12-h dark cycle and had free access to food and water. All experiments and procedures about mice were approved by the Institutional Animal Care and Use Committee.

### Cdr1as KO Mice

The Cdr1as KO mice were generated and maintained on the C57BL/6J background with CRISPR/Cas9-mediated genome editing technology. The sgRNAs (sgRNA1 and sgRNA4) to mouse Cdr1as and Cas9 mRNA were co-injected into fertilized mouse eggs to generate targeted KO offspring. F0 founders were identified by PCR followed by sequence analysis, which were bred to WT mice to test germline transmission and F1 animal generation.

### Fundus Photography and High-Resolution Spectral-Domain Optical Coherence Tomography (SD-OCT)

Cdr1as KO and C57BL/6J mice were anesthetized intraperitoneally with pentobarbital sodium. Before the examination, 2.5% hydroxypropyl methylcellulose was dropped into the eyes to improve the connection with the machine (Micron IV, Phoenix Research Labs), and then fundus photography was performed. For SD-OCT measurements, images crossing through the optic nerve were obtained and collected for each eye. The thicknesses of the different retinal layers were measured using Insight software (Pleasanton, CA, United States).

### Electroretinography (ERG)

Electroretinography responses in both eyes of mice were carried out as described in the instrument manual (Phoenix Research Laboratories) and as previously described (Jin et al., 2014; Xiang et al., 2017). In brief, mice were dark-adapted overnight and then anesthetized intraperitoneally with pentobarbital sodium. Pupils were dilated with 0.5% tropicamide. A drop of 1% methylcellulose was applied on the cornea to improve the conjunction with the gold wire loop electrode. Ground electrodes and referential needles were punctured into the tail and scalp, respectively. Scotopic ERG was recorded at −2.2 and 0.3 log cd-s/m^2^ stimulus intensity with a 30-s interstimulus interval. Photopic ERG was measured at 0.65 log cd-s/m^2^ with a 0.4-s interstimulus interval after 10 min of light adaptation with a background illumination of 30 cd/m^2^.

### Immunofluorescence Staining

Whole eyeballs of mice were extracted immediately after euthanasia. After removing the cornea and lens, the eyecups were fixed in 4% paraformaldehyde for 2 h. Then, retinas were dehydrated in 30% (wt/vol) sucrose and then embedded in embedding medium (Neg-50, Thermo). Sections with 12-μm-thick cryosection slides were cut and washed with PBS, blocked in blocking buffer [4% bovine serum albumin (BSA), 0.5% Triton X-100 in PBS] for 1 h, treated with primary antibody at 4°C overnight, and then incubated with secondary antibody at room temperature for 1 h. The following primary and secondary antibodies were used: mouse anti-Rhodopsin (1:500, Sigma), rabbit anti-Cone-arrestin (1:50, Millipore), mouse anti-Calbindin (1:200, BD), rabbit anti-Recoverin (1:500, Millipore), mouse anti-α-synuclein (1:200), guinea pig anti-Vglut1 (1:200, Millipore), rabbit anti-Pkcα (1:100, Sigma), mouse anti-Ctbp2 (1:100, BD Biosciences), mouse anti-Ctbp2 (1:200, BD), rabbit anti-Pax6 (1:200, Sigma), mouse anti-Rbpms(1:50, Santa Cruz) and donkey anti-rabbit IgG conjugated to Alexa Fluor 488 (1:200, Life Technologies), donkey anti-rabbit IgG conjugated to Alexa Fluor 594 (1:200, Life Technologies), donkey anti-mouse IgG conjugated to Alexa Fluor 594 (1:200, Li-cor Biosciences), and goat anti-guinea pig IgG conjugated to Alexa Fluor 568 (1:200, Abcam). Nuclei were stained with 4, 6-diamidino-2-phenylindole (DAPI, 1:3000, Invitrogen, Carlsbad, CA, United States), Morphologies of the stained retina were imaged using a Leica SP8 laser scanning confocal microscope (Leica, Wetzlar, Germany).

### *In situ* Hybridization

BaseScope is an efficient method of ISH. ACdr1as probe targeting the junction site was designed and BaseScope^TM^ Reagent Kit—RED supplied by Advanced Cell Diagnostics (ACD) was used. Slides of mouse retinas were dried completely at RT and treated with hydrogen peroxide for 10 min and then protease plus for another 30 min at room temperature; then, probe hybridization was performed strictly according to the manufacturer’s protocol. Images were acquired using a microscope (Nikon Eclipse).

### Visual Evoked Potential (VEP)

Visual evoked potential was recorded using the same equipment as ERG. After 5 min of dark adaptation in cages, the mice were anesthetized as previously described. Pupils were dilated with 0.5% tropicamide. A drop of 1% methylcellulose was applied on the cornea to avoid eye drying. During each VEP session, body temperature was maintained at 37°C using a heating pad. The active electrode was positioned on the back of the head at the location of the visual cortex; the reference electrode was put into the cheek, and the ground electrode was placed into the tail. VEPs were evoked by continuousflash of 1.4 and 2.0 Hz, 5 cds/m^2^ white light. VEP signals were recorded by a commercial system (RETIport, Roland Consult GmbH, Germany).

### Optomotor Response (OMR)

Mice were placed on a raised platform surrounded by a motorized drum with vertical black and white stripes; the drum could rotate clockwise or anticlockwise. The stripe pattern slowly rotated around the animal at a speed of 12°/s, and triggered the optomotor reflex. Animal behavior was monitored by camera, and the behavior was automatically detected and then analyzed by OptoDrum software (Striatech, Germany). The stimulus pattern was continuously and automatically adjusted during the experiment to find the Cdr1as KO and WT mouse visual thresholds (visual acuity or contrast sensitivity).

### RNA Isolation, qRT-PCR, and Small RNA Sequencing

The retinas were isolated from mice and collected in TRIzol (Invitrogen, United States). RNAs were extracted using the RNeasy Kit (Qiagen). For miRNA, qRT-PCR was carried out using the Bulge-Loop miRNA qRT-PCR Starter Kit according to the manufacturer’s protocol. For mRNAs, complementary DNA (cDNA) was synthesized using random primers (Promega) and quantified by FastStart Universal SYBR Green Master Mix (Roche). GAPDH was used as the reference gene. Primers for genes were listed in Supplementary Table 3. For small RNA sequencing, retinas were isolated from 2M WT and Cdr1as KO mice (*n* = 3); the integrity and quantity of RNA was assessed using Agilent 2200 TapeStation and Qubit2.0, respectively. Small RNA libraries were constructed and sequenced by HiSeq 2500 (Illumina, United States) at Ribobio Co. Ltd. (Ribobio, China). miRDeep2 was used to identify known mature miRNA based on miRBase21 and predict novel miRNAs. The expression levels of miRNAs were normalized by RPM, RPM = (number of reads mapping to miRNA/number of reads in clean data) × 10^6^. Differential expression between WT and Cdr1as KO retina was calculated by edge R algorithm according to the criteria of | Fold Change| ≥ 1.5 and *P*-value < 0.05.

### GO and KEGG Pathway Analysis

GO and KEGG pathway analysis was based on the targets of different expressed 25 miRNAs in Cdr1as KO retina. The target genes of miRNAs were predicted using seven tools, including PITA^1^, RNA22^2^, miRNAmap^3^, microT^4^, miRanda^5^, PicTar^6^, and TargetScan^7^. Target genes predicted by at least three tools and verified by CLIP assay (Zhou et al.; Li et al., 2014) were retained for further GO and KEGG pathway analysis^8^.

### Western Blots

Retinas (P300) were isolated, collected, and lyzed in lysis buffer containing 1× PMSF. Protein was then extracted and quantified using a BCA protein assay kit (Invitrogen). Proteins were separated using SDS-PAGE and then analyzed by anti-α-synuclein (1:2000, Abcam), anti-phospho-α-synuclein (1:2000, Wako), and anti-GAPDH (1:1000, KangChen Biotech).

### Statistical Analysis

The values shown in the graphs represent averages of several independent experiments and the actual number of samples for each experiment stated in the figure legends. The results are represented as mean ± SEM. The statistical significance was assessed by a two-tailed student’s *t*-test. ^∗^*P* < 0.05; ^∗∗^*P* < 0.005; ^∗∗∗^*P* < 0.001.

## Data Availability Statement

The original contributions presented in the study are included in the article/Supplementary Material. Further inquiries can be directed to the corresponding author.

## Ethics Statement

The animal study was reviewed and approved by the Institutional Animal Care and Use Committee WMU.

## Author Contributions

Z-BJ conceived and supervised the whole study. X-JC, M-LL, Y-HW, HM, ZW, X-YW, and C-JZ performed the experiments. XX participated in the data interpretation. SB, Z-HX, and HZ performed bioinformatics statistical analysis. X-JC wrote the manuscript. Z-BJ revised the manuscript. All authors contributed to the article and approved the submitted version.

## Conflict of Interest

The authors declare that the research was conducted in the absence of any commercial or financial relationships that could be construed as a potential conflict of interest.

## Footnotes

1 http://genie.weizmann.ac.il/pubs/mir07/mir07data.html

2 http://cbcsrv.watson.ibm.com/rna22.html

3 http://mirnamap.mbc.nctu.edu.tw/

4 http://www.microrna.gr/microT

5 http://www.microrna.org/microrna/home.do

6 http://www.pictar.org/

7 http://www.targetscan.org/

8 https://david.ncifcrf.gov/summary.jsp
