# Supplementary material for: Abundant Neural circRNA Cdr1as Is Not Indispensable for Retina Maintenance
Source: Front Cell Dev Biol. 2020 Nov 6;8:565543. doi: 10.3389/fcell.2020.565543 (PMC7677238; doi:10.3389/fcell.2020.565543)
Supplement: Supplementary file 1 [file Table_1.docx]

**Supplementary materials**


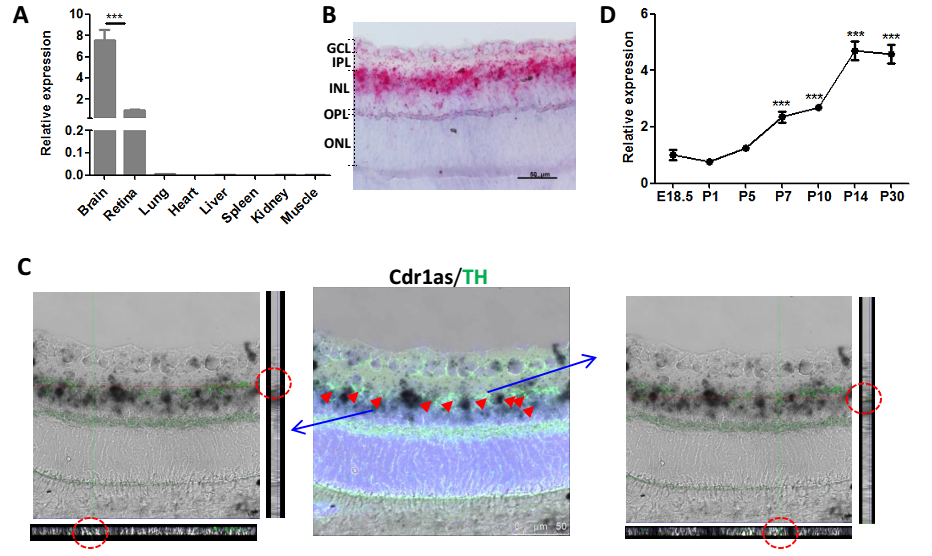


**Figure S1 Expression pattern of Cdr1as in retina and other tissues.**

A: Expression pattern of Cdr1as in different tissues of WT mouse (n=3). B: Expression and location of Cdr1as in WT mouse retinas were analyzed using BaseScope assay. C: Cdr1as expression pattern during retinal development (n=3). D: Co-localization of Cdr1as and TH in Cdr1as and WT retinas at P70.


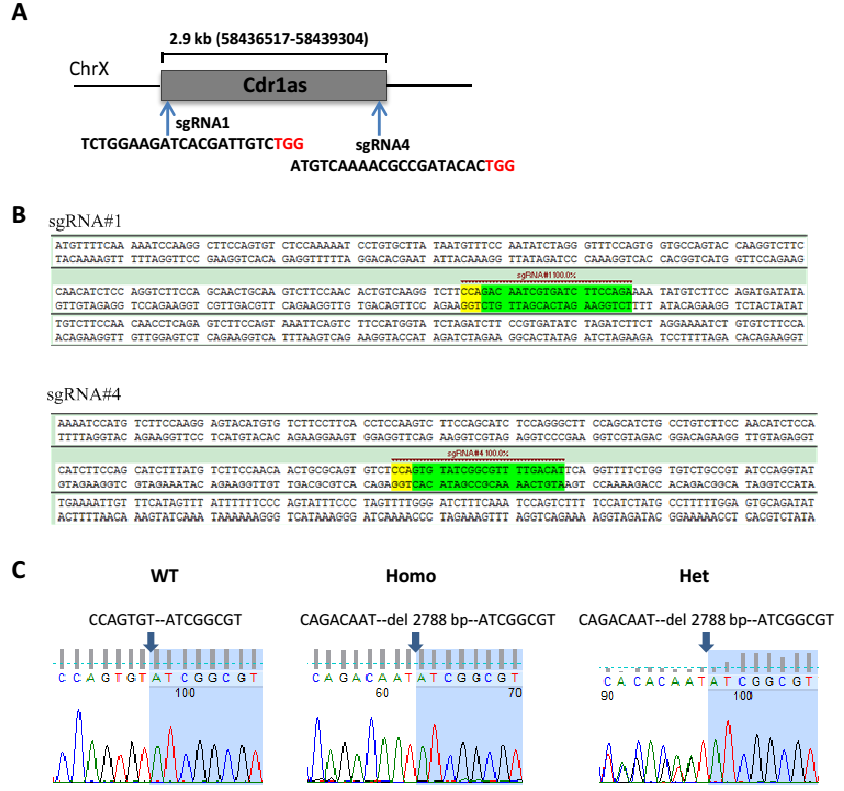


**Figure S2** A: Cdr1as was removed using CRISPR/Cas9. B: The highlighted area shows the two Cas9/sgRNA-targeting sites. PAM sequences were highlighted in yellow. C: Head to tail junction sequences of Cdr1as in WT, het and homo retinas based on Sanger sequencing.


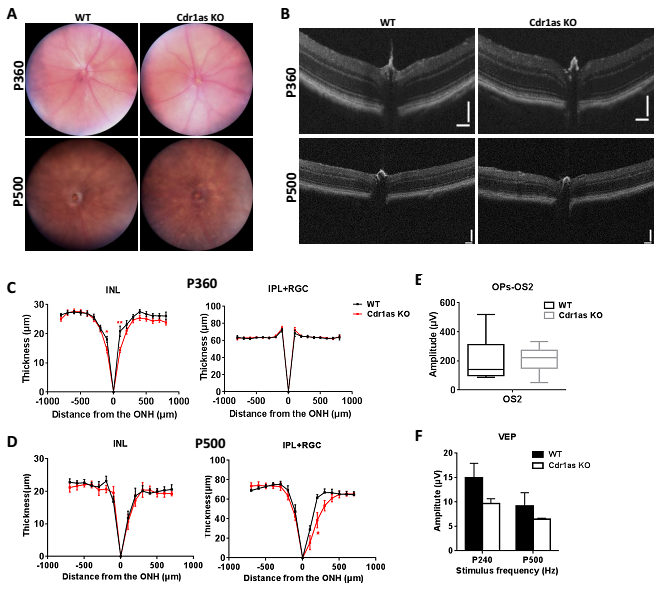


**Figure S3 Evaluation of retinal structure and function at aged Cdr1as KO mice**

A: Fundus photographs of WT and Cdr1as KO retinas at P360 (n=4) and P500 (n=6). B: OCT examination of WT and Cdr1as KO retinas at P330 and P500. C, D: Quantification of the thickness of the INL and IPL+RGC from the SD-OCT image in B (n=8 for P360, n=6 for P500). E: OPS in Cdr1as KO and WT retinas at P240 (n=8 for WT, n=8 for KO). F: VEP recording of Cdr1as KO and WT retinas at P240 (n=5) and P500 (n=3).


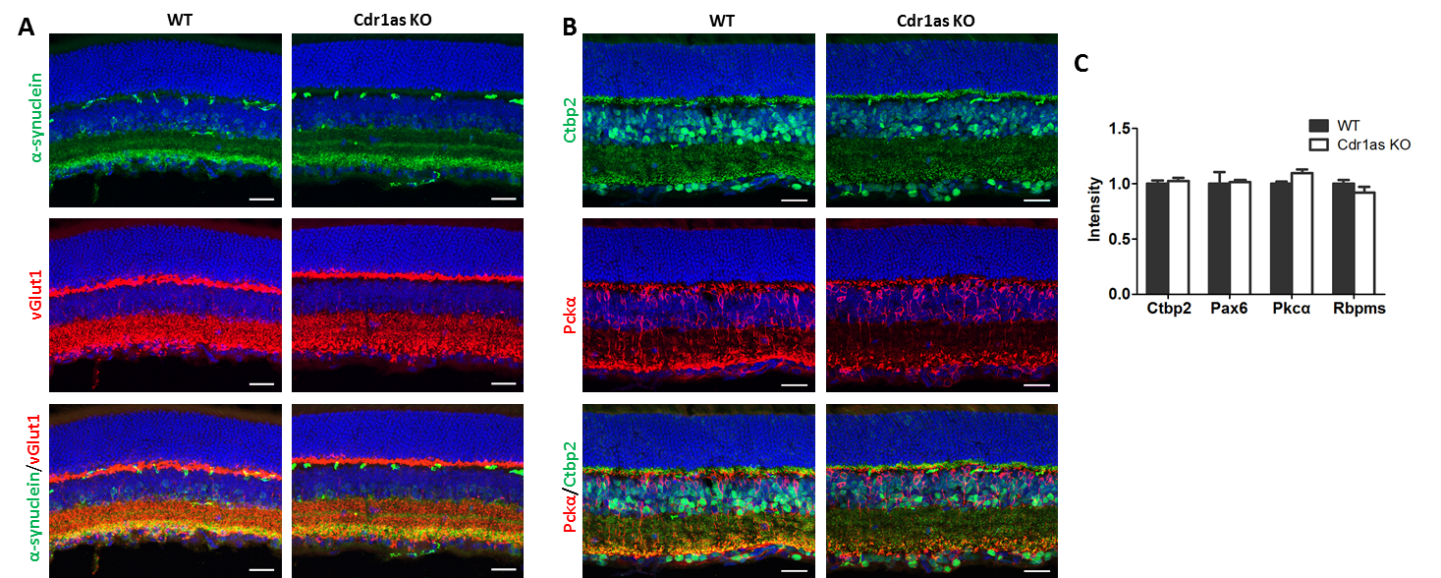


Figure S4 A, B: Immunostaining of α-synuclein/vGlut1 (A) and pkcα/Ctbp2 (B) in WT and Cdr1as KO retinas (n=3, Scale bar: 25 μm). C: Quantification of Rbpms-, Pax6-, Pcka- and Ctbp2-positive signals in immunostaining assay.


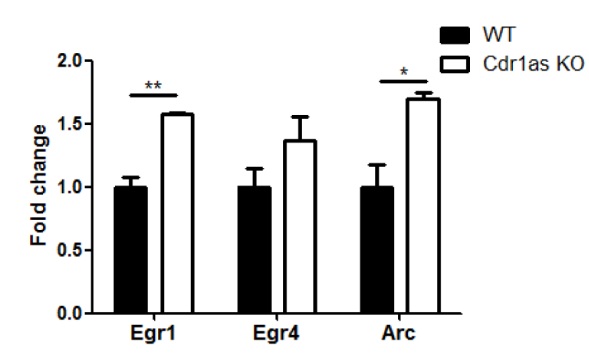


**Figure S5 Expression patterns of IEGs in Cdr1asKO and WT retinas (n=3).**


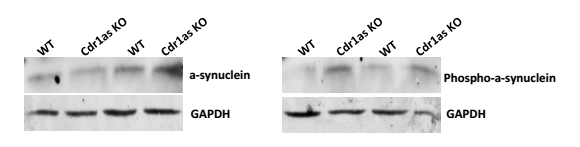


**Figure S6** **Changs in protein level of α-synuclein and phosphor-α-synuclein in Cdr1as and WT retinas at P300.**

**Table S1. sgRNAs were designed for Cdr1as KO using CRISPR-cas9 strategy**

| **gRNA** | **Sequence** |
| --- | --- |
| sgRNA1  (matching reverse strand of gene): | TCTGGAAGATCACGATTGTCTGG |
| sgRNA4  (matching reverse strand of gene): | ATGTCAAAACGCCGATACACTGG |

**Table S2. Primer pairs for genotyping were designed to differentiate mutants from wild-type mice**

| **Primer** | **Sequence** |
| --- | --- |
| F1 | CTAGGTCTCTTTATCTCTAGGG |
| R1 | GCTTGAGCATAACTGACTACCA |
| R5 | CGGAAGATCTAGATACCATGG |

**Table S3: Primers used in qRT-PCR**

| **Gene** | **Forward primer** | **Reverse primer** |
| --- | --- | --- |
| Nr4a3 | CACACTCACTGTCCTTCCTTCA | AACACAACCCCAACACGATACT |
| Klf4 | GGCCCAACTACCCTCCTTTC | TAGTCACAAGTGTGGGTGGC |
| Irs2 | AACCTGAAACCTAAGGGACTGG | CGGCGAATGTTCATAAGCTGC |
| α-Synuclein | TCGCTCTTGCCACACCTAGCT | GCCCATCAGTGACGTGAGGTG |
| c-Fos | TACTACCATTCCCCAGCCGA | ACTTCTCAGCAGCCTTGAGAC |
| Egr4 | CTCCACCTGAGCGACTTCTC | GCTCAAAGCCCAGCTCAAGA |
| Egr1 | TTCAATCCTCAAGGGGAGCC | TAACTCGTCTCCACCATCGC |
| Arc | CCCCAGCAGTGATTCATACCA | ACTTCTCAGCAGCCTTGAGAC |
| GAPDH | AGGTCGGTGTGAACGGATTTG | TGTAGACCATGTAGTTGAGGTCA |
